# Locomotor sensitization to EtOH: contribution of β-Endorphin

**DOI:** 10.3389/fnmol.2012.00087

**Published:** 2012-08-29

**Authors:** Stephani Dempsey, Judith E. Grisel

**Affiliations:** Furman UniversityGreenville, SC, USA

**Keywords:** alcohol, EtOH, knockout, mu-receptor, opioid, transgenic

## Abstract

Alcohol use disorders, like all drug addictions, involve a constellation of adaptive changes throughout the brain. Neural activity underlying changes in the rewarding properties of alcohol reflect changes in dopamine transmission in mesolimbic and nigrostriatal pathways and these effects are modulated by endogenous opioids such as β-Endorphin. In order to study the role of β-Endorphin in the development of locomotor sensitization to repeated EtOH exposure, we tested transgenic mice that vary in their capacity to synthesize this peptide as a result of constitutive modification of the *Pomc* gene. Our results indicate that mice deficient in β-Endorphin show attenuated locomotor activation following an acute injection of EtOH (2.0 g/kg) and, in contrast to wildtype mice, fail to demonstrate locomotor sensitization after 12 days of repeated EtOH injections. These data support the idea that β-Endorphin modulates the locomotor effects of EtOH and contributes to the neuroadaptive changes associated with chronic use.

## Introduction

Alcohol use disorders are a worldwide concern, devastating the health of individuals, families, and communities. Like virtually all disorders involving behavior, alcoholism results from a rich interaction of environmental influences expressed through genetic propensities, which makes it difficult to understand particular causal mechanisms. One strategy frequently employed by those seeking to elucidate biological substrates of complex traits is to utilize animal models in which simpler components of the phenotype are isolated and explored in a controlled laboratory setting (Crabbe, 2012).

One consequence of exposure to all addictive drugs is the ability to activate neural substrates involved in reward. At the heart of these circuits is the mesolimbic pathway which, when stimulated, leads to dopamine release in the nucleus accumbens. This pathway conveys information about stimulus salience. Another major dopaminergic circuit mediates locomotion, and all addictive drugs also stimulate movement. Thus, dopamine activity alerts an organism to important stimuli and motivates behavior; these pathways are both turned on by addictive substances (Robinson and Berridge, 1993; Kalivas and Volkow, 2005; Sanchis-Segura and Spanagel, 2006).

While the depressant effects of alcohol (EtOH) are widely appreciated, administration of lower doses, or analysis during the absorptive phase of blood-EtOH curve produce reliable stimulant effects, particularly in individuals susceptible to abuse and addiction (e.g., Wise, 1987; Phillips and Shen, 1996). Furthermore, chronic exposure to EtOH can result in sensitization to these changes, defined as an increase in behavioral stimulation following repeated administration, and this is also heritable. For instance, some inbred strains of mice are more prone to locomotor activation and sensitization than others (Phillips et al., 1995). Other strains of mice have been selectively bred to model these effects (Phillips et al., 1991; Crabbe et al., 1992). Moreover, studies have found genetic correlations between effects of EtOH on locomotor activity and measures of EtOH reinforcement (Malila, 1978; Phillips et al., 1998; Ponomarev and Crabbe, 2002).

At least one way that EtOH activates the neural pathways involved in reward and movement is through its ability to stimulate the synthesis and release of the opioid peptide β-Endorphin (β-E; Schulz et al., 1980; Gianoulakis, 1990, 2009; Scanlon et al., 1992; Przewlocka et al., 1994; Froehlich et al., 2000). β-Endorphin modulates dopamine activity in the mesolimbic pathway (Widdowson and Holman, 1992; Oswald and Wand, 2004; Zapata and Shippenberg, 2006; Jarjour et al., 2009) as well as in the nigrostriatal pathway (Willis, 1987; Boyadjieva and Sarkar, 1994; Sanchis-Segura and Aragon, 2002; Jarjour et al., 2009; Lam et al., 2010). Genetic differences in these opioid circuits correlate with a liability for alcohol use disorders in humans (i.e., Topel, 1988; Gianoulakis et al., 1989, 1996; Froehlich et al., 2000). In a series of studies, Sanchis-Segura and colleagues have made a strong case that β-E in the arcuate nucleus of the hypothalamus mediates EtOH induced locomotor activation and we and others have shown that low opioid activity compromises the reinforcing effects of EtOH (Grisel et al., 1999; Roberts et al., 2000; Racz et al., 2008).

Though the particular alleles and gene products contributing to EtOH induced locomotor changes remain obscure (Phillips et al., 1995), opioid peptides have been implicated (Prunell et al., 1987; Kuribara et al., 1991; Sanchis-Segura and Aragon, 2002; Sanchis-Segura et al., 2005). In this study we evaluated the effect of β-E on the development of locomotor sensitization to repeated EtOH exposure using transgenic mice that vary in their capacity to synthesize the peptide. “Knockout” (KO) mice have a premature stop codon inserted into their *Pomc* gene and therefore fail to produce β-E. These mice are fully backcrossed onto the C57BL/6J strain, which provide a useful comparison along with heterozygote (HET) mice that have 50% of control levels of the endogenous opioid.

## Materials and methods

### Subjects

Subjects were adult naïve male and female wildtype controls (C57BL/6J; B6), β-E deficient (B6.129S2-*Pomc*^*tm1Low*^/J; KO), and heterozygous (HT) mice. Transgenic mice were developed over a decade ago in the laboratory of Malcolm Low (Rubinstein et al., 1996) by insertion of a premature stop codon into the *Pomc* gene and have been fully backcrossed onto the B6 genome. Homozygotes (KO) cannot synthesize β-E, though all other *Pomc* products show normal expression. Opioid receptor expression also remains unchanged (Rubinstein et al., 1996). HT mice produce 50% of B6 levels of β-E.

Mice for these studies were bred from isogenic pairs derived in-house from HT progenitors purchased from Jackson Laboratories (Bar Harbor, ME, USA). The gene mutation has been fully backcrossed to the C57BL/6J strain (>20 generations). Mice were group housed by sex with 4–5 per Plexiglas cage following weaning at 20–21 days and maintained in a at 21 ± 2°C colony room on a reverse 12:12 light:dark cycle with lights on at 7 PM. Water and food were available *ad libitum*.

### Experimental protocol

We followed the method developed in Tamara Phillips' lab (Lessov et al., 2001) in which C57BL/6J mice evince robust locomotor sensitization following repeated administration of EtOH, although these investigators suggests that at least some of the increased locomotor activity on test day may reflect a “novelty response” (Meyer et al., 2005) since in this paradigm the mice have not been in the test chamber for several days before the sensitization measure is taken.

On Days 1–3 and 14 of the two-week protocol, mice were assayed during the dark phase of their light/dark cycle in a Plexiglas open field arena (50 cm^3^) equipped with infrared sensors and coupled to Tru Scan software (Coulbourn Instuments, Whitehall, PA). Horizontal distance traveled and the number of rears (two front feet off the ground) was assessed for each mouse during the 10 min test period on Day 1–3 and 14. The cage floor was thoroughly cleaned between experimental subjects with non-toxic, low-odor solution, and testing order was randomized across genotypes.

On Days 1–3 animals received injections and were placed in the testing arena for 10 min. On Day 1 and Day 2 all animals received saline but on Day 3, two groups of animals—designated chronic or acute EtOH (CE or AE) received 2.0 g/kg EtOH instead of saline. Days 4-13, animals were injected and then immediately placed back in their home cages; there was no measurement of locomotor activity. The CE group got 2.5 g/kg EtOH each of these days, and the AE and chronic saline (CS) groups got equivolume saline. On Day 14, all animals received 2.0 g/kg EtOH and locomotor activity was evaluated for 10 min. All injections were delivered intraperitoneally (i.p.) and EtOH was administered in a 20% (vol:vol) solution in saline. All procedures were carried out in accordance with the National Institutes of Health guidelines and approved by the Animal Care and Use Committee of Furman University.

### Statistical analysis

Data were analyzed using a three factor ANOVA with genotype (three levels: B6, HT, and KO), experimental condition (AE, CE, and CS), and sex for main effects, on days 1–3 and 14 separately. In addition, a repeated measure ANOVA was conducted across days by strain and condition (excluding sex). Significant interactions were further examined using Fisher's least significant difference (LSD) test. Statistical analyses were performed using SPSS Statistics 17.0. The criterion for significance was set at *p* ≤ 0.05.

## Results

There were main effects of sex on virtually every measure, as females are well known to have more locomotor activity under basal conditions as well as following EtOH administration (e.g., Lynn and Brown, 2009; Tayyabkhan et al., 2002). However, in this overall analysis, there were no significant interactions involving sex and genotype—i.e., the strain differences were not dependent upon sex, and therefore data were collapsed across sex for analysis.

**Figure 1:**
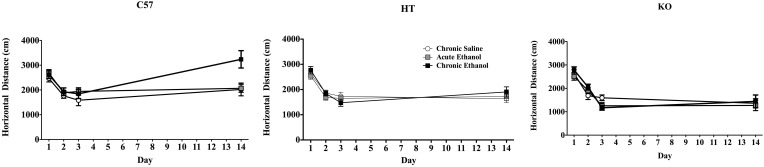
**Locomotor activity in mice during four, 10 min test periods on Days 1–3 and 14 of a 2-week experimental protocol.** On Days 1 and 2 all mice received intraperitoneal saline injections, on Day 3 the chronic saline (CS) group received saline again, but all other subjects received 2.0 g/kg EtOH. On Days 4–13, half of these were administered 2.5 g/kg EtOH (Chronic EtOH—CE) and the remainder was given equivolume saline (Acute EtOH—AE, and CS) and all were placed in their home cages immediately following injection. On Day 14, all animals were administered 2.0 g/kg EtOH and evaluated in the activity chambers. The *left panel* shows the horizontal distance traveled in cm (data show mean ± SE) of adult male and female C57BL/6J mice, demonstrating the development of locomotor sensitization in CE-treated subjects. The *middle panel* shows analogous experimental groups of heterozygote (HT) mice and the *right panel* shows the same data in β-E deficient (KO) mice. Neither of these latter two groups developed locomotor sensitization.

In the repeated measures ANOVA there was a main effect of strain on horizontal distance traveled [*F*_(2, 91)_ = 7.265, *p* < 0.001] but no main effect of experimental condition [*F*_(2, 91)_ = 2.25, *p* > 0.05] or interaction between these two variables [*F*_(4, 91)_ = 1.002, *p* > 0.05]. There was also a main effect of test day *F*_(3, 273)_ = 94.306, *p* < 0.001, interactions between strain and day *F*_(6, 273)_ = 10.584, *p* < 0.001, and strain and experimental condition *F*_(6, 273)_ = 3.634, *p* < 0.01 as well as a triple interaction between strain, day and drug condition *F*_(12, 273)_ = 2.061, *p* < 0.05. While B6 mice in the CE condition (both sexes) showed sensitization, neither of the β-endorphin deficient groups did (Figure 1).

There was no difference in distance traveled or rears on Day 1 across either lines or drug groups (though all received saline on Day 1) and no interactions. With one exception the results were the same on Day 2 as on Day 1: there was no difference in distance traveled across genotypes or experimental groups and no interactions. However, there was a significant effect of genotype on rears on Day 2 in that KO did not habituate as readily as either B6 or HT mice [*F*_(2, 91)_ = 7.911, *p* < 0.01; data not shown].

**Figure 2:**
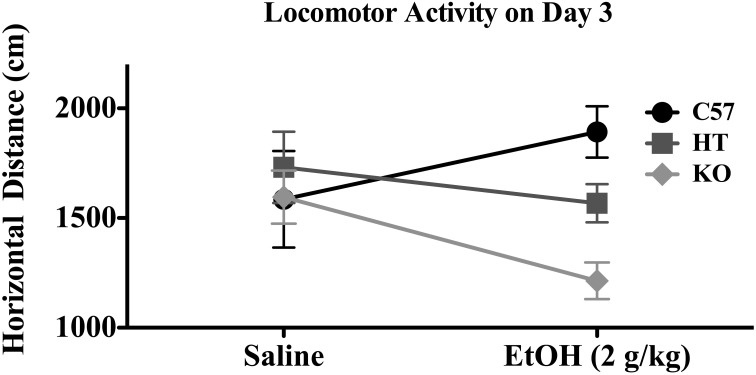
**Horizontal distance traveled in mice of each genotype on Day 3 of the experimental protocol.** Mice were either given 2.0 g/kg EtOH by intraperitoneal injection or equivolume saline immediately before 10 min evaluation (see text for experimental detail). Data represent group means ± SE.

On Day 3, there was a significant effect of genotype on distance traveled [*F*_(2, 91)_ = 6.377, *p* < 0.01], evincing a direct relationship between β-E levels and horizontal distance traveled, but in this Two-Way ANOVA with 3 treatment groups and 3 genetic lines, there was no main effect of treatment or interaction between treatment and genotype. However, because the AE and CE groups were treated identically and injected with 2.0 g/kg EtOH, they were collapsed into a general “EtOH” group and compared to saline-treated subjects in a separate 2-factor ANOVA (this time with only two levels of treatment) evaluating locomotion on Day 3 (horizontal distance in cm). As shown in Figure 2, B6 tended to be stimulated by EtOH, while KOs were sedated by EtOH and HTs were intermediate. This is substantiated in the statistical results in which there was a main effect of strain: [*F*_(2, 91)_ = 3.750, *p* < 0.05], but not of drug [*F*_(1, 91)_ = 0.593, *p* > 0.05] but there was an interaction between strain and drug [*F*_(2, 91)_ = 3.750, *p* < 0.05]. While there was no effect of genotype on rears, there was an effect of EtOH [*F*_(2, 91)_ = 166.636, *p* < 0.001] in which the drug generally decreased rearing activity, but this measure on Day 3 did not depend upon strain.

On Day 14 there were differences between genotype in the distance traveled [*F*_(2, 91)_ = 20.356, *p* < 0.001], differences across treatment groups [*F*_(2, 91)_ = 4.222, *p* < 0.05], and a significant interaction between genotype and treatment group [*F*_(4, 91)_ = 2.961, *p* < 0.05]: post-hoc analysis indicated sensitization only in B6 mice (in the CE group; see Figure 1). There were no differences in rears between strain or treatment groups and no significant interaction between these two measures on Day 14.

**Figure 3:**
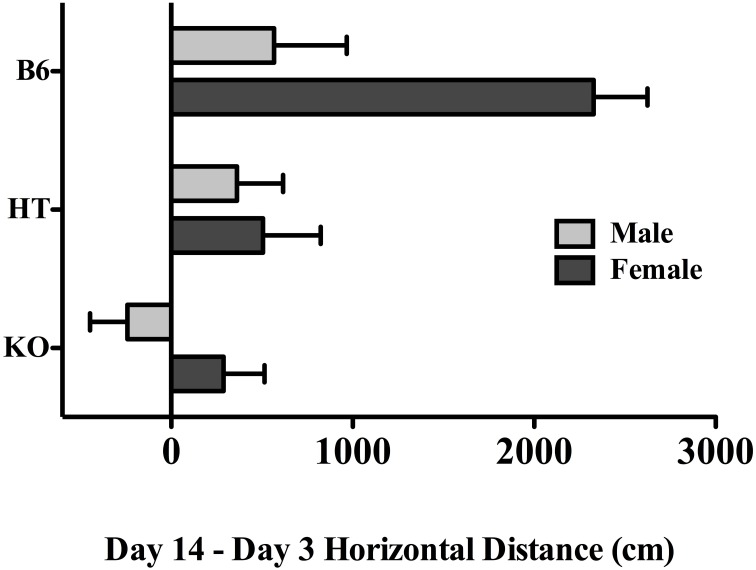
**The difference in horizontal locomotor activity between the first and last exposure to EtOH in chronically treated mice across the three genotypes and both sexes (see text for experimental detail).** Data represent group means ± SE.

In order to directly compare the magnitude of locomotor sensitization that developed across the experimental period while taking into account the genotypic differences on Day 3 (see above) we conducted a 2-way (strain and sex) planned comparison within the CE treatment groups, using a difference score that was calculated by subtracting horizontal activity on Day 3, after the first exposure to EtOH, from Day 14 activity, following the chronic regimen. Here, the effect of genotype was significant with *F*_(2, 29)_ = 9.260, *p* < 0.001, indicative of a positive correlation between β-E levels and locomotor activity on Day 14. The post-hoc test revealed that B6 mice differed from either of the β-E deficient lines. There was, as in the overall analysis, a main effect of sex [*F*_(1, 29)_ = 8.726, *p* < 0.01], but also, a (just) significant interaction between strain and sex [*F*_(2, 29)_ = 3.312, *p* = 0.051]. These change scores are depicted separately by strain and sex in Figure 3, where evidence of locomotor plasticity is more evident in wildtype females than in all other groups.

## Discussion

Mice deficient in β-Endorphin demonstrate a blunted locomotor response to acute alcohol, and also fail to develop locomotor sensitization after 12 days of daily drug administration. Though there were no observable differences in activity under baseline conditions (following injection with saline) 2.0 g/kg EtOH moderately stimulated locomotor activity in C57BL/6J mice but depressed it in mice lacking β-E. Furthermore, while repeated injections of EtOH led to locomotor sensitization in C57BL/6J mice, transgenics with either low or absent β-E showed no evidence of this plasticity. These data replicate findings by others showing the development of sensitization in this inbred strain (e.g., Lessov et al., 2001; Tarragón et al., 2012) and extend them by suggesting that β-E plays a critical role in this change as transgenic subjects unable to synthesize β-E, but otherwise virtually identical to controls, fail to demonstrate this plasticity.

Drug sensitization is thought to underlie changes associated with alterations in the reward pathway such as drug-induced conditioned place preference, operant self-administration and other forms of drug seeking (Vezina, 2004). Indeed, a empirical evidence and theoretical explorations link the neural plasticity underlying locomotor sensitization to the behavioral characteristics of drug abuse including drug seeking, compulsive use, and relapse (Robinson and Berridge, 1993, 2008; Kalivas et al., 2005). Thus, many have argued that behavioral sensitization to the locomotor effects of drugs provides an index of neural changes mediating the dependent state (Robinson and Berridge, 1993; Kalivas and Volkow, 2005; Sanchis-Segura and Spanagel, 2006). The present findings support the contention that the opioid peptide β-E plays a critical role in the neural substrates of alcohol reinforcement and addiction. Along this line, EtOH self-administration in animals depends upon this peptide (Grisel et al., 1999; Williams et al., 2007; Racz et al., 2008; but also see Grahame et al., 1998) and clinical studies have associated β-E levels with liability toward alcohol use disorders (Gianoulakis et al., 1989; Wand et al., 2001; Zalewska-Kaszubska and Czarnecka, 2004).

As with all behavior, the neural mechanisms of sensitization are complex and multidimensional. Though the current study, along with previous reports (Camarini et al., 2000; Pastor and Aragon, 2006; Abrahao et al., 2008; Tarragón et al., 2012) strongly implicates endorphins, these peptides are surely not acting alone. For instance, both endorphins and endomorphins are highly efficacious agonists at μ receptors, and though these receptors appear to be unchanged in transgenic mice (Rubinstein et al., 1996), other opioids may also contribute. Moreover, evidence supports the involvement of various other neurotransmitters including amino acids (i.e., γ-aminobturic acid and glutamate) and monoamines, in this plasticity (Broadbent et al., 1995; Chester and Cunningham, 1999; Meyer and Phillips, 2007; Carrara-Nascimento et al., 2011). Repeated EtOH administration has also been linked to activation of the Hypothalamic Pituitary Adrenal (HPA) axis and shown to be dependent upon the neuroendocrine response to stress (Roberts et al., 1995; Pastor et al., 2008, 2012).

β-E is involved in a wide array of behaviors, including many of those associated with analgesia, reward, attachment, and stress. While activation of the stress (HPA) axis leads to synthesis and release of β-E, this peptide plays a role in endocrine and behavioral allostasis. Exposure to a stressor induces the hypothalamus to secrete corticotropin releasing hormone (CRH) in the adenohypophysis, mounting a neuroendocrine response, and leading to activation of the sympathetic nervous system and behavior. Upon stimulation by CRH, the anterior pituitary turns on *POMC* transcription to stimulate synthesis of adrenocorticotrophin hormone (ACTH) and β-E (among others). ACTH leads to the synthesis and release of glucocorticoids from the adrenal gland but may also inhibit *POMC* activity (Suda et al., 1988, 1993). Negative feedback is typical in the stress response, and virtually every stress-induced chemical change subsequently contributes to dampening further HPA activation. For example, corticosterone (in rodents) or cortisol (in humans), through interaction with a dense population of glucocorticoid receptors in the brain, suppresses HPA activity. Blockade or deletion of either CRH or glucocorticoids prevents the acquisition of EtOH-induced locomotor sensitization (Roberts et al., 1995; Pastor et al., 2008, 2012) suggesting that an intact neuroendocrine response to stress is necessary to exhibit locomotor sensitization to EtOH.

These data are somewhat contradictory because, although synthesized and released in response to stress, β-E inhibits the stress axis by counteracting CRH synthesis in the paraventricular nucleus of the hypothalamus (Buckingham, 1986; Plotsky, 1991; Hunt and Zakhari, 1995; Janssen and Arntz, 2001; Amat et al., 2005; Ribeiro et al., 2005). Thus, low or absent β-E is associated with exaggerated neuroendocrine and behavioral responses to stress (Buckingham, 1986; Grisel et al., 2008; Barfield et al., 2010) and disruptions in coping behavior (Hunt and Zakhari, 1995; Gianoulakis, 1998; Barry and Grisel, 2012). We have shown, i.e., an inverse relationship between β-E levels and anxious behavior, as well as adrenal weight, in these mice (Grisel et al., 2008). Since low β-E compromises the ability to manage stressful stimuli (Gianoulakis, 1998; Sarkar et al., 2007; Barfield et al., 2010) one might expect augmented, rather than attenuated, locomotor sensitization in β-E deficient mice. However, because CRH mediates the EtOH-induced increase in β-E (Gianoulakis, 1998; Lam and Gianoulakis, 2011) perhaps the low CRH activity indirectly affects sensitization, through a consequent blunting of the β-E surge following EtOH administration.

It is well documented that acute exposure to EtOH, like exposure to stressors, causes the synthesis and release of β-E. The relationship between opioids and EtOH-induced locomotor changes has been extensively studied by Carlos Aragon and his colleagues, in over two decades of research. Early studies implicated the opioid system in the effects of stress and EtOH on movement (Aragon et al., 1990; Trudeau et al., 1991). Fifteen minutes of restraint stress decreased locomotor activity, but this effect of stress was blocked (in an opioid-dependent manner) by EtOH pre-treatment. These data fit with the recent findings (Pastor et al., 2012) that CRH and corticosterone are necessary to evince EtOH-induced locomotor changes in mice. However, this group also showed that either pharmacologic antagonism of μ-opioid receptors or lesioning endorphinergic neurons in the hypothalamus prevents EtOH-induced increases in locomotor activity and other forms of adaptation including conditioned place preference (Sanchis-Segura et al., 2000; Sanchis-Segura and Aragon, 2002; Pastor et al., 2004, 2005; Pastor and Aragon, 2008). The endogenous opioid system has been implicated in several aspects of the rewarding and addictive actions of ethanol. Modulation of the mesolimbic dopamine system by β-E contributes to positive reinforcement following drug administration (see Roth-Deri et al., 2008 for review). Individual variation in the β-E response to EtOH has been used to explain differences in the liability toward high consumption and abuse (Gianoulakis, 1996, 1998; Froehlich et al., 2000; Dai et al., 2002, 2005). The current study, demonstrating a failure to develop locomotor sensitization in an animal model of endorphin deficiency, adds to the growing body of pre-clinical research suggesting that β-E modulates the neuroplasticity underlying chronic changes in behavior associated with the development of alcohol addiction.

### Conflict of interest statement

The authors declare that the research was conducted in the absence of any commercial or financial relationships that could be construed as a potential conflict of interest.
